# Specific disruption of Lnk in murine endothelial progenitor cells promotes dermal wound healing via enhanced vasculogenesis, activation of myofibroblasts, and suppression of inflammatory cell recruitment

**DOI:** 10.1186/s13287-016-0403-3

**Published:** 2016-10-28

**Authors:** Jun Hee Lee, Seung Taek Ji, Jaeho Kim, Satoshi Takaki, Takayuki Asahara, Young-Joon Hong, Sang-Mo Kwon

**Affiliations:** 1 Department of Pharmacology and Toxicology, University of Alabama at Birmingham School of Medicine, Birmingham, AL 35294 USA; 2 Department of Physiology, Laboratory for Vascular Medicine and Stem Cell Biology, Medical Research Institute, School of Medicine, Pusan National University, Yangsan, 626-870 Republic of Korea; 3 Research Institute of Convergence Biomedical Science and Technology, Pusan National University School of Medicine, Yangsan, Republic of Korea; 4 Department of Immune Regulation, Research Centre for Hepatitis and Immunology, Research Institute, National Centre for Global Health and Medicine, Chiba, Japan; 5 Department of Regenerative Medicine Science, Tokai University School of Medicine, Kanagawa, Japan; 6 Division of Cardiology of Chonnam National University Hospital, Cardiovascular Convergence Research Center Nominated by Korea Ministry of Health and Welfare, Gwangju, 501-757 Republic of Korea

**Keywords:** Endothelial progenitor cell, Wound healing, Neovascularization, Anti-inflammatory, Cell-based therapy

## Abstract

**Background:**

Although endothelial progenitor cells (EPCs) contribute to wound repair by promoting neovascularization, the mechanism of EPC-mediated wound healing remains poorly understood due to the lack of pivotal molecular targets of dermal wound repair.

**Methods and Results:**

We found that genetic targeting of the *Lnk* gene in EPCs dramatically enhances the vasculogenic potential including cell proliferation, migration, and tubule-like formation as well as accelerates in vivo wound healing, with a reduction in fibrotic tissue and improved neovascularization via significant suppression of inflammatory cell recruitment. When injected into wound sites, *Lnk* ^-/-^ EPCs gave rise to a significant number of new vessels, with remarkably increased survival of transplanted cells and decreased recruitment of cytotoxic T cells, macrophages, and neutrophils, but caused activation of fibroblasts in the wound-remodeling phase. Notably, in a mouse model of type I diabetes, transplanted *Lnk* ^-/-^ EPCs induced significantly better wound healing than *Lnk* ^+/+^ EPCs did.

**Conclusions:**

The specific targeting of Lnk may be a promising EPC-based therapeutic strategy for dermal wound healing via improvement of neovascularization but inhibition of excessive inflammation as well as activation of myofibroblasts during dermal tissue remodeling.

## Background

Wound repair is a complex but well-organized biological process that requires the coordinated action of various cell types and multiple signaling cascades [1, 2]. The process consists of three phases: inflammation; tissue formation including proliferation, angiogenesis, and granulation; and tissue remodeling [1]. In the inflammation phase, leukocytes including neutrophils and macrophages are recruited to the wound site to eliminate some pathogens and cell debris. In the phase of new tissue formation, several types of cells migrate and proliferate. Keratinocytes migrate to the wound site, and engrafted endothelial cells (ECs) or endothelial progenitor cells (EPCs) form new blood vessels. Fibroblasts also migrate from adjacent tissues and produce the extracellular matrix (ECM). In the tissue-remodeling phase, the ECM is remodeled by fibroblasts, and myofibroblasts play a role in connective tissue compaction and wound contraction [3, 4]. In several pathological conditions such as diabetes and chronic diseases, impairment of these well-ordered healing processes leads to a delay or overhealing of the wound, resulting in a functional disorder, pain, infection, or fibrosis. To address these problems, several researchers have suggested possible strategies to make wounds more regenerative than scar forming, e.g., by applying small molecules, biomimetic scaffolds, gene therapy, electrical manipulation, or a stem/progenitor cell-based therapy [3, 5–7].

EPCs are a promising cell source for treatment of ischemic diseases. Since EPCs were isolated from adult peripheral blood [8], they have been found to migrate to the injury site and contribute to new-vessel formation as well as to play a pivotal role in vascular maintenance [9]. Although a transplant of EPCs into an ischemic tissue dramatically enhances the tissue repair process, pathological conditions, including inflammation, ischemia, and nutrient deficiency, lower the efficacy of engraftment and decrease survival rates of EPCs in injured tissue. To enhance the therapeutic efficacy, several studies describe key strategies where molecular targeting of EPCs facilitates their functionality and therapeutic effects at an injury site [6, 10, 11]. For example, transfer of the manganese superoxide dismutase gene into EPCs increases wound healing in a mouse model of type 2 diabetes [10], and modulation of the CCL5–CCR5 interaction enhances wound tissue repair through recruitment of EPCs [6]. In addition, a CXCR4 antagonist, AMD 3100, promotes wound healing by mobilizing bone marrow (BM)-derived EPCs to an injury site [11]. Nevertheless, the mechanism of EPC-mediated wound healing remains poorly understood due to the lack of key molecular targets of dermal wound repair.

Lnk adaptor protein (SH2B3) is a member of the SH2B family of adaptor proteins, which are implicated in regulation and modulation of various cell signaling pathways [12]. Lnk participates in the major signaling pathways, including those related to interleukin (IL)-3, stem cell factor (SCF)/c-Kit, thrombopoietin (TPO)/myeloproliferative leukemia protein (MPL), erythropoietin (EPO)/EPO receptor (EPOR), platelet-derived growth factor (PDGF)/PDGF receptor (PDGFR), tumor necrosis factor (TNF), and integrins [12]. In addition, Lnk affects several effector targets, such as phosphoinositol-4,5-bisphosphate 3-kinase (PI3K)/Akt, p38 mitogen-activated protein kinases (MAPK), extracellular signal-regulated kinases (ERK1/2), and Janus kinase 2 (JAK2)/signal transducer and activator of transcription 3 (STAT3), and STAT5 [12]. Our previous studies showed that Lnk deficiency enhances the capacity for cell growth, endothelial commitment, mobilization, and recruitment of EPCs [13]. Moreover, selective downregulation of Lnk in EPCs promotes vascular repair and neovascularization in a murine model of hindlimb ischemia through regulation of the JAK2/STAT3 axis [14].

In the present study, our aim was to test whether Lnk-deficient EPCs promote wound healing through augmentation of EPC bioactivities and neovascularization with activation of myofibroblasts as well as suppression of inflammatory cell recruitment at wound sites in wound-healing models and in a murine model of type 1 diabetes. This report shows that the specific targeting of Lnk may be an effective EPC-based therapeutic strategy for promotion of dermal wound healing.

## Methods

### Animals

The *Lnk* ^*-/-*^ mice were generated as previously reported [15]. Experiments were performed on 8-week-old male C57BL/6 mice (Biogenomics, Seoul, Korea) and *Lnk* ^*-/-*^ mice maintained in a 12-hour light/dark cycle in accordance with the regulations of Pusan National University. The protocols were approved by the Institutional Animal Care and Use Committee of Pusan National University School of Medicine, on the basis of the Guide for the Care and Use of Laboratory Animals.

### Murine BM-derived EPC culture

Isolation of BM-derived EPCs was performed as previously reported [13]. BM mononuclear cells (MNCs) isolated from tibia and femur of wild-type and *Lnk* ^*-/-*^ mice were plated in cell culture dishes coated with 1 % gelatin (Sigma-Aldrich, St. Louis, MO, USA) at the density of 5 × 10^5^/cm^2^ and were cultured with endothelial basal medium 2 (EBM-2; Lonza, Walkersville, MD, USA) supplemented with 5 % fetal bovine serum (FBS; Lonza) to obtain the EPC-enriched population. The cells were placed in a humidified incubator at 37 °C and 5 % CO_2_. After 4 days, nonadherent cells were discarded, and a fresh culture medium was added. Cultures were maintained for another 3 days to obtain the putative EPCs.

### The murine model of streptozotocin-induced diabetes

To induce diabetes, a single high dose of streptozotocin (STZ; 225 mg/kg; Sigma-Aldrich) was intraperitoneally injected into C57BL/6 mice (fasted for 16 h beforehand, body weight 20–23 g). Every week after STZ administration, serum glucose levels were measured using an Accu-Check Advantage glucometer (Roche, Indianapolis, IN, USA) during nonfasting status. Mice with a plasma glucose level >200 mg/dl at 3 weeks after injection were regarded as having STZ-induced diabetes [16].

### The wound-healing model

The excisional wound model was generated as described previously [17]. In brief, after shaving and cleaning with 70 % ethanol, the dorsal skin of wild-type or *Lnk* ^*-/-*^ mice (*n* = 5 per group) was picked up at the midline and two layers of skin were perforated with a sterile disposable biopsy punch (4 mm diameter; Miltex, York, PA, USA), generating one wound on each side of the midline. After establishing the excisional wound model, the process of wound healing was observed for 10 days. In EPC transplantation experiments, after establishing the excisional wound model, wild-type or *Lnk* ^*-/-*^ EPCs (10^5^ cells) in 80 μl of PBS or 80 μl of PBS alone were homogeneously administered into the subcutaneous tissue around the wound defect in normal mice or in mice with STZ-induced diabetes (*n* = 5 per group). Each wound site was digitally photographed at the indicated time points after injury, and wound areas were determined by tracing the wound margins using the Image J software (http://rsbweb.nih.gov/ij/). The wound area at each time point was calculated as a percent area of the original wound.

### Histological and immunohistological analysis

The wounds were excised with the surrounding tissue. The tissue samples were fixed with 4 % paraformaldehyde in PBS at 4 °C for 24 h and embedded in paraffin to prepare histological or immunohistological slides. For histological analysis, tissue slices were stained with hematoxylin and eosin (H&E) or Masson’s trichrome dye. For immunohistological analysis, the slices were incubated with anti-CD31, proliferating cell nuclear antigen (PCNA), cleaved caspase 3, alpha-smooth muscle actin, and vimentin antibodies (all from Santa Cruz Biotechnology, Dallas, TX, USA) and followed by incubation with Alexa Fluor 488- or 594-conjugated secondary antibodies (Thermo Fisher Scientific, Waltham, MA, USA). Nuclei were stained with 4′,6-diamidino-2-phenylindole (Sigma-Aldrich). Immunostained slides were examined under confocal microscopy (Olympus, Tokyo, Japan). Each experiment was repeated at least three times.

### Flow cytometric analysis

To verify recruitment of the EPC population or leukocytes, wound tissues were harvested and digested with 0.1 % type II collagenase (Sigma-Aldrich) after postoperative day 3 or 7. EPCs or single cells derived from wound tissues were subjected to flow cytometric analysis using anti-Sca-1, anti-c-Kit, anti-Flk-1, anti-CD34, anti-CD3, anti-CD8, anti-CD11, and anti-CD45 antibodies (all from BD, San Jose, CA, USA). Flow cytometry was performed using a fluorescence-activated cell sorter (FACS; BD). Histograms represent the cell number (y-axis) versus the fluorescence intensity (x-axis, log scale). FACS gating was performed using cells stained with isotype-matched IgG as a negative control. For each antibody, the proportion of positively stained cells was determined by comparison with isotype-matched control cells. The percentage of positively stained cells is indicated by the positive peaks. Red lines indicate cells stained with each antibody, and black lines indicate the negative control cells. Each experiment was repeated at least three times.

### The cell proliferation assay

Cell proliferation was assessed using the BrdU Cell Proliferation Assay Kit (Cell Signaling Technology, Beverly, MA, USA) or Ez-CYTOX Kit (Daeil Biotech, Suwon, South Korea) according to the manufacturer’s instructions. Each experiment was repeated at least three times.

### Tubule-like formation assay

To assess tubule-like formation capacity of EPCs, a Matrigel tube formation assay was performed. Matrigel (BD) was added to 96-well plates and incubated at 37 °C. Cells (10^4^/well) were seeded in Matrigel-coated plates and incubated for 6 h at 37 °C and 5 % CO_2_. The cells were monitored by phase contrast microscopy (Olympus). Each experiment was repeated at least three times.

### The migration assay

Cells were plated in 6-well plates and grown until confluence and then the monolayer was wounded with a cell scraper. The detached cells were removed by gentle washing with the medium. Cells were incubated for 24 h at 37 °C and 5 % CO_2_ and examined under a microscope (Olympus) equipped with a × 40 objective lens. Each experiment was repeated at least three times.

### Statistical analysis

All data are expressed as mean ± SEM. One-way analysis of variance was used followed by Tukey’s post hoc test for multiple comparisons, or a Student’s *t* test was used for paired comparisons. A *p* value < 0.05 was considered to indicate a significant difference.

## Results

### Improved wound healing under the influence of enhanced engrafted EPCs in *Lnk*^*-/-*^ mice

**Fig. 1:**
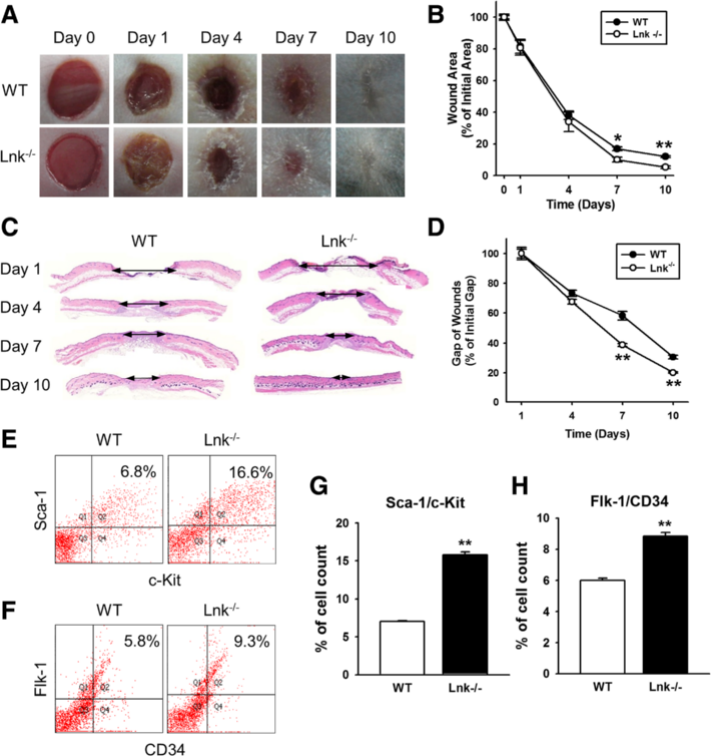
Lnk deficiency improves wound repair in a murine model of an excisional wound. **a** Photographs of the wound were captured on days 0–10 after administration of an excisional wound to wild-type (WT) and Lnk-deficient mice. **b** This graph shows the proportion of the wound area at the indicated time points post wounding. Values are mean ± SEM; ^*^ *p* < 0.05 and ^**^ *p* < 0.01 compared to the wound area in Lnk-deficient mice. **c** An H&E-stained section of a skin wound in WT and Lnk-deficient mice at the indicated time points post wounding. **d** The graph shows the proportion of the wound gap at the indicated time points post wounding. Values are mean ± SEM; ^**^ *p* < 0.01 compared to the wound gap in WT mice. **e** Wound sites were analyzed to identify Sca-1/c-Kit-positive EPCs by FACS analysis. **f** Wound sites were analyzed to determine Flk-1/CD34-positive EPCs by FACS analysis. **g** and **h** The graph shows the percentage of Sca-1/c-Kit-positive cells (**g**) and Flk-1/CD34-positive cells (**h**) at wound sites of WT and Lnk-deficient mice. Values are mean ± SEM; ^**^ *p* < 0.01 compared to WT mice

Our previous studies showed that in vivo genetic targeting of Lnk enhances osteogenesis, neovascularization, and astrogliosis in mouse models of some diseases [13, 18, 19]. To test whether the lack of the *Lnk* gene affects wound healing in an in vivo murine excisional wound model, we generated an excisional wound in *Lnk* ^+/+^ and *Lnk* ^-/-^ mice (Fig. 1a). Wound closure was significantly enhanced in *Lnk* ^-/-^ mice compared with wild-type mice (Fig. 1b). Histological analysis by H&E staining showed that the gap of wounds was significantly decreased in *Lnk* ^-/-^ mice compared with wild-type mice (Fig. 1c and d). To confirm that the EPC population is involved in wound healing, after digestion of wound tissues, isolated cells were characterized by flow cytometric analysis for Sca-1^+^/c-Kit^+^ markers and Flk-1^+^/CD34^+^ markers, which represent typical EPC population markers (Fig. 1e and f). FACS analysis indicated that Sca-1^+^/c-Kit^+^ and Flk-1^+^/CD34^+^ cells were significantly more prevalent in wound tissues of *Lnk* ^-/-^ mice than in wound tissues of wild-type mice (Fig. 1g and h). These results suggest that the specific disruption of the *Lnk* gene promotes wound repair in an excisional wound model through the recruitment of EPC populations to ischemic sites.

### The enhanced vasculogenic potential of Lnk-deficient EPCs

**Fig. 2:**
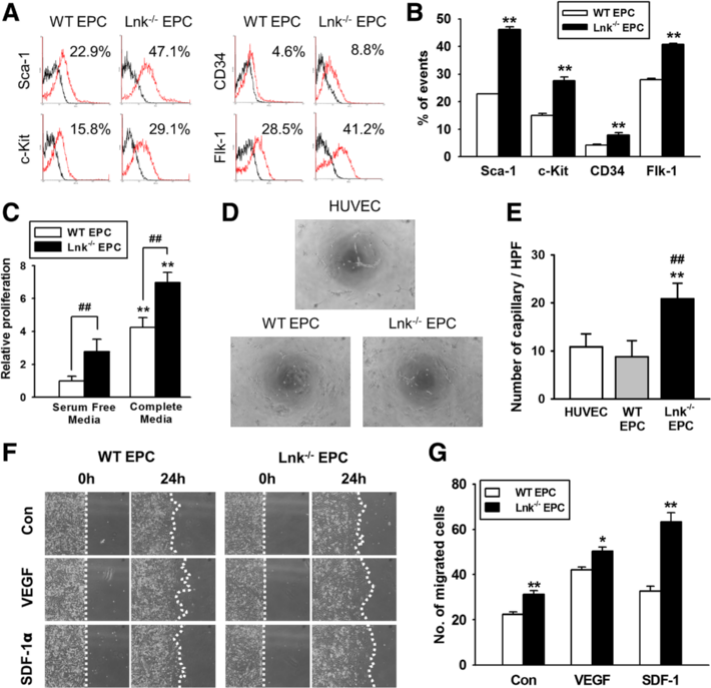
Evaluation of characteristics and functionalities of EPCs. **a** After isolation of EPCs from wild-type (WT) and Lnk-deficient mice, EPC surface markers, including Sca-1, c-Kit, CD34, and Flk-1, were analyzed on a FACS. **b** The graph shows the percentage of EPCs with surface markers among WT and Lnk-deficient EPCs. Values are mean ± SEM; ^**^ *p* < 0.01 compared to WT EPCs. **c** Proliferation of EPCs was evaluated in serum-free or complete media by a 5-bromo-2′-deoxyuridine (BrdU) assay. Values are mean ± SEM; ^**^ *p* < 0.01 compared to proliferation of WT EPC in a serum-free medium; ^##^ *p* < 0.01 compared to WT EPCs. **d** Tube formation capacity of HUVECs, WT EPCs, and Lnk-deficient EPCs was determined by a Matrigel tube formation assay (magnification × 40). **e** The graph shows the number of capillaries among HUVECs, WT EPCs, and Lnk-deficient EPCs. Values are mean ± SEM; ^**^ *p* < 0.01 compared to HUVECs and ^##^ *p* < 0.01 compared to WT EPCs. **f** Migration capacity was assessed by a wound scratch assay (magnification × 40). **g** The graph shows the number of migrating cells among WT EPCs and Lnk-deficient EPCs in response to VEGF or SDF-1α. Values are mean ± SEM; ^**^ *p* < 0.01 compared to WT mice

To evaluate EPC surface markers, we isolated BM-derived EPCs from *Lnk* ^+/+^ and *Lnk* ^-/-^ mice. Interestingly, typical murine EPC markers including Sca-1, c-Kit, CD34, and Flk-1, were significantly upregulated in Lnk-deficient EPCs in comparison with wild-type EPCs (Fig. 2a and b). To further assess EPC bioactivities, we confirmed cell proliferation, tubule-like formation, and migration capacity. Proliferation was significantly increased in Lnk-deficient EPCs, compared with wild-type EPCs in both a serum-free medium and complete medium (Fig. 2c). The Matrigel tube formation assay revealed that Lnk-deficient EPCs have higher tube formation capacity than wild-type EPCs do (Fig. 2d and e). Migration capacity was also significantly increased in Lnk-deficient EPCs compared with wild-type EPCs in response to vascular endothelial growth factor (VEGF) and stromal cell-derived factor 1 (SDF-1) (Fig. 2f and g). These findings indicated that the lack of the *Lnk* gene in a BM niche gives rise to functional EPCs because of expression of typical EPC surface markers and because of enhanced EPC bioactivities, including cell proliferation, cell migration, and tubule-like formation.

### Improved wound repair after subcutaneous injection of Lnk-deficient EPCs

**Fig. 3:**
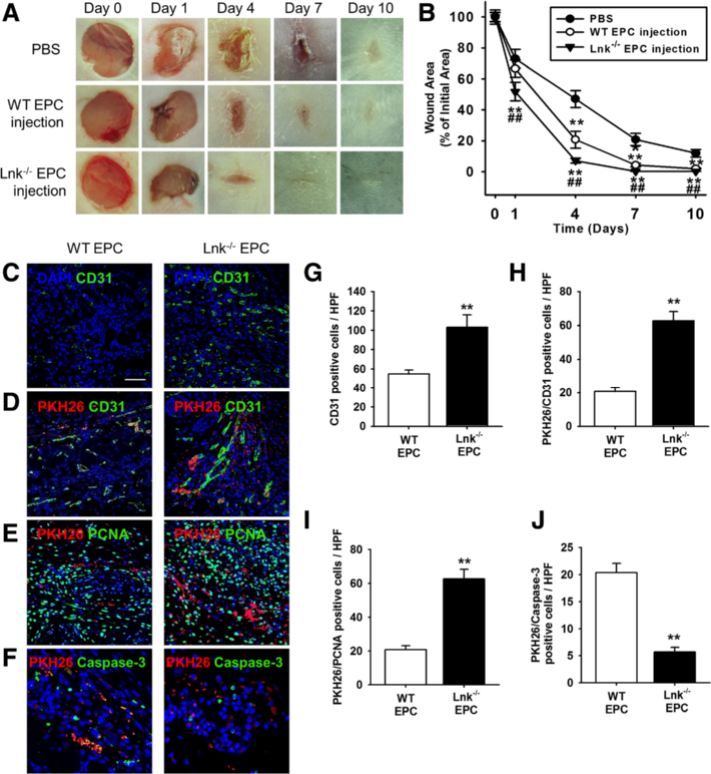
Assessment of functional recovery in a wound excision model after subcutaneous injection with EPCs. **a** After administration of an excisional wound to wild-type (WT) mice, we subcutaneously transplanted PBS, WT EPCs, and Lnk-deficient EPCs into wound sites. **b** The graph shows the proportion of the wound area at the indicated time points post wounding. Values are mean ± SEM; ^**^ *p* < 0.01 compared to injection with PBS and ^##^ *p* < 0.01 compared to injection with WT EPCs. **c** On postoperative day 10, capillary formation was evaluated by immunofluorescence staining for CD31 (*green*). Nuclei were stained with DAPI (*blue*). Scale bar = 50 μm. **d** After PKH26 dye (*red*)-stained EPCs were subcutaneously injected into wound sites, incorporation of the transplanted EPCs into vessels was assessed by immunofluorescent staining for CD31 (*green*) on postoperative day 3. **e** Proliferation of transplanted EPCs was confirmed by immunofluorescent staining for PCNA (*green*) on postoperative day 3. **f** Apoptosis among transplanted EPCs was detected by immunofluorescent staining for cleaved caspase 3 (*green*) on postoperative day 3. **g** The graph shows the number of CD31-positive cells. Values are mean ± SEM; ^**^ *p* < 0.01 compared to injection with WT EPCs. **h** The graph shows the number of PKH26/CD31-positive cells. Values are mean ± SEM; ^**^ *p* < 0.01 vs. injection with WT EPCs. **i** The graph shows the number of PKH26/PCNA-positive cells. Values are mean ± SEM; ^**^ *p* < 0.01 compared to injection with WT EPCs. **j** The graph shows the number of PKH26/cleaved caspase 3-positive cells. Values are mean ± SEM; ^**^ *p* < 0.01 compared to injection with WT EPCs

To explore the effects of Lnk-deficient EPCs on wound repair in a murine excisional wound model, after creation of excisional wounds in wild-type mice, we subcutaneously injected wild-type or Lnk-deficient EPCs into the wound border area (Fig. 3a). The wound area was significantly reduced by injection of Lnk-deficient EPCs, as compared with the area after injection of PBS or wild-type EPCs (Fig. 3b). On postoperative day 10, neovascularization was assessed by immunofluorescence staining for CD31 (Fig. 3c). This staining indicated that neovascularization was significantly enhanced by injection of Lnk-deficient EPCs as compared with injection of wild-type EPCs (Fig. 3g). To verify incorporation into the vessels, cell proliferation, and survival of the transplanted EPCs, after membranes of EPCs were labeled with PKH26, the cells were transplanted into wound border sites. Three days after the transplant of EPCs, incorporation into vessels as well as cell proliferation and apoptosis were assessed by immunofluorescent staining for PKH26/CD31 (incorporation; Fig. 3d), PKH/PCNA (proliferation; Fig. 3e), and PKH26/cleaved caspase 3 (apoptosis; Fig. 3f). Incorporation into vessels and proliferation were significantly increased in transplanted Lnk-deficient EPCs, compared with wild-type EPCs (Fig. 3h and i). Apoptosis was significantly decreased in transplanted Lnk-deficient EPCs compared with wild-type EPCs (Fig. 3j). These results suggest that Lnk-deficient EPCs improve wound healing through enhancement of neovascularization and via augmentation of incorporation into vessels, proliferation, and survival of the transplanted cells.

### Decreased numbers of inflammatory cells among engrafted Lnk-deficient EPCs

**Fig. 4:**
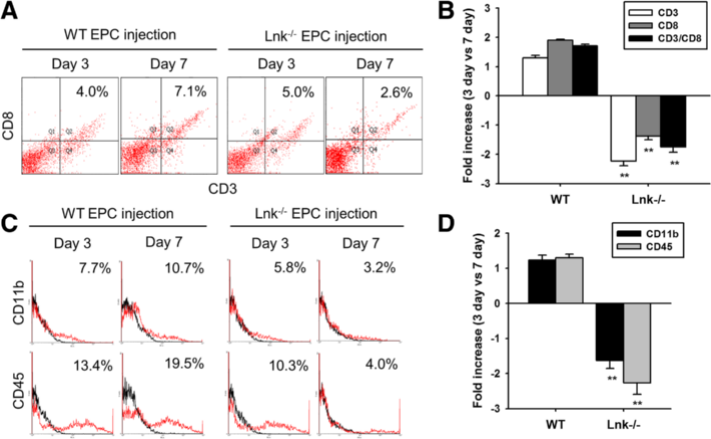
A transplant of Lnk-deficient EPCs suppresses the recruitment of inflammatory cells. After injection of wild-type (WT) and Lnk-deficient EPCs into wound sites, wound tissues were analyzed to determine the recruitment of cytotoxic T cells (CD3- and CD8-positive cells), macrophages (CD11b-positive cells), and neutrophils (CD45-positive cells) on postoperative days 3 and 7. **a** The recruitment of cytotoxic T cells in wound tissues was assessed by FACS analysis. **b** The percentage of CD3/CD8 double-positive cells on postoperative days 3 and 7. Values are mean ± SEM; ^**^ *p* < 0.01 compared to postoperative day 3, respectively, and ^##^ *p* < 0.01 compared to injection with WT EPCs. **c** The recruitment of macrophages and neutrophils to wound tissues was assessed by FACS analysis. **d** The percentage of CD11b- and CD45-positive cells on postoperative days 3 and 7. Values are mean ± SEM; ^**^ *p* < 0.01 compared to postoperative day 3, respectively, ^#^ *p* < 0.05 and ^##^ *p* < 0.01 compared to injection with WT EPCs

In the inflammatory phase (1–3 days after injury), leukocytes are recruited to the wound site to remove cell debris and pathogens. On the other hand, persistent presence of inflammatory cells at wound sites leads to delayed wound healing and to cell death [20]. To confirm the inhibitory effect of Lnk-deficient EPCs on the recruitment of inflammatory cells to wound sites 3 days after injury, we subcutaneously injected wild-type and Lnk-deficient EPCs and then assessed the recruitment of inflammatory cells to wound sites on postoperative day 7. The percentage of CD3 and CD8 double-positive cells, which are a cytotoxic T cell population, was significantly decreased after injection of Lnk-deficient EPCs as compared with injection of wild-type EPCs (Fig. 4a and b). In addition, the number of cells positive for CD11b (a macrophage marker) and CD45 (a neutrophil marker) was significantly decreased after injection of Lnk-deficient EPCs on postoperative day 7, as compared with that after injection of wild-type EPCs (Fig. 4c and d). These data suggested that a transplant of Lnk-deficient EPCs suppresses the recruitment of inflammatory cells in the proliferation or remodeling phase.

### Activated fibroblasts among engrafted Lnk-deficient EPCs

**Fig. 5:**
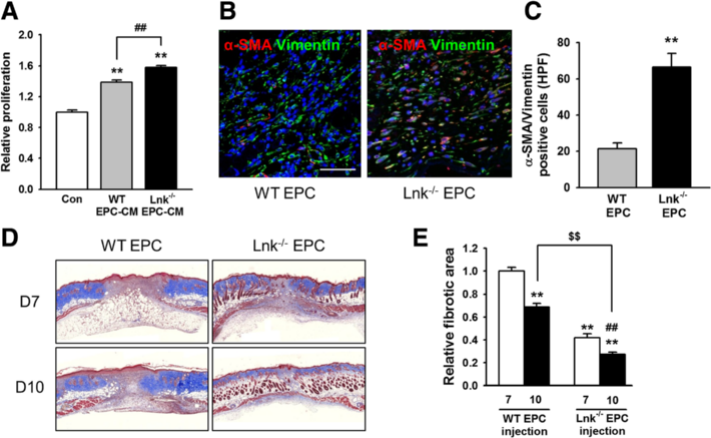
The cross-talk of EPCs with fibroblasts in vitro and in vivo. **a** After isolation of skin fibroblasts from wild-type mice, these cells were cultured in a WT EPC conditioned medium (CM) or Lnk-deficient EPC CM for 24 h. Proliferation of fibroblasts was assessed by the BrdU assay. Values are mean ± SEM; ^**^ *p* < 0.01 compared to control, and ^##^ *p* < 0.01 compared to WT EPC-CM. **b** After subcutaneous injection with EPCs into the wound area, the presence of myofibroblasts (α-SMA and vimentin double-positive cells) at wound sites was evaluated by immunofluorescent staining for α-SMA (*red*) and vimentin (*green*) on postoperative day 7. Nuclei were stained with DAPI (*blue*). Scale bar = 50 μm. **c** The graph shows the number of α-SMA and vimentin double-positive cells at wound sites on postoperative day 7. Values are mean ± SEM; ^**^ *p* < 0.01 vs. injection with WT EPCs. **d** Masson’s trichrome staining was performed to determine the fibrotic area on postoperative days 7 and 10. **e** The graph shows the relative fibrotic area 7 and 10 days after EPC injection. Values are mean ± SEM; ^**^ *p* < 0.01 compared to injection with WT EPC on postoperative day 7, ^##^ *p* < 0.01 compared to injection with Lnk-deficient EPCs on postoperative day 7, and ^$$^ *p* < 0.01 compared to injection with WT EPCs on postoperative day 10

In the remodeling phase, activated fibroblasts play an important role in tissue remodeling and wound repair [3]. To determine whether Lnk-deficient EPCs activate fibroblasts and induce differentiation of fibroblasts into myofibroblasts, we assessed proliferation of fibroblasts in vitro and differentiation of fibroblasts into myofibroblasts in vivo. After addition of an EPC-conditioned medium to fibroblasts, dermal-fibroblast proliferation was confirmed. Proliferation of fibroblasts was significantly facilitated by conditioned media from Lnk-deficient EPCs in contrast to wild-type EPC conditioned media (Fig. 5a). Seven days after injection of EPCs into the wound area of wild-type mice, myofibroblast differentiation was assessed by immunofluorescent staining for alpha-smooth muscle actin (α-SMA) and vimentin (Fig. 5b). The number of myofibroblasts that were α-SMA and vimentin double-positive was significantly increased by injection with Lnk-deficient EPCs, as compared with injection of wild-type EPCs (Fig. 5c). Masson’s trichrome staining indicated that injection of Lnk-deficient EPCs significantly decreased the fibrotic area on postoperative days 7 and 10 in comparison with the injection of wild-type EPCs (Fig. 5d and e). These results suggest that Lnk-deficient EPCs promote wound healing through activation of fibroblasts.

### Wound repair is improved by the engrafted Lnk-deficient EPCs in a model of a type I diabetes excisional wound

**Fig. 6:**
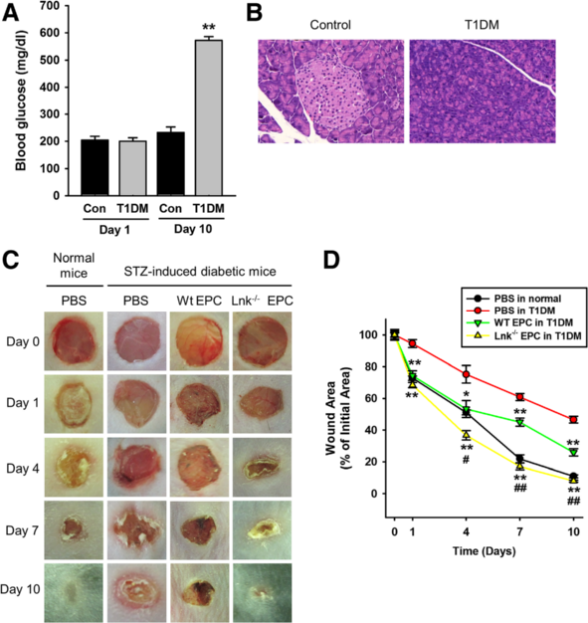
Effects of a Lnk-deficient EPC transplant on wound repair in mice with STZ-induced diabetes. **a** To establish a mouse model of type 1 diabetes mellitus (T1DM), blood glucose was assessed after a streptozotocin (STZ) injection. **b** H&E staining of a tissue slice shows necrosis of β-cells in the pancreas (magnification × 40). **c** After injection of EPCs into an excisional wound of normal mice or mice with STZ-induced diabetes, photographs of the wound were captured on days 0–10. **d** The graph shows the proportion of the wound area at the indicated time points post wounding. Values are mean ± SEM; ^*^ *p* < 0.05 and ^**^ *p* < 0.01 compared to injection with PBS in mice with STZ-induced diabetes, ^#^ *p* < 0.05 and ^##^ *p* < 0.01 compared to injection of WT EPCs into mice with STZ-induced diabetes

To test whether a transplant of Lnk-deficient EPCs improves wound healing in a mouse model of type 1 diabetes, we created a murine model of STZ-induced diabetes (Fig. 6a and b). After administering an excisional wound to mice with STZ-induced diabetes, wild-type or Lnk-deficient EPCs were transplanted into the wound border area (Fig. 6c). The wound healing area was significantly increased in the “Lnk-deficient EPC injection” group compared with the other groups (Fig. 6d). These results indicated that Lnk-deficient EPCs improved wound healing in the model of type 1 diabetes.

## Discussion

The wound repair process is a complex and well-coordinated regenerative response that involves a cross-talk among several types of cells, growth factors, cytokines, ECM, and soluble factors. Various risk factors, however, such as diabetes, hypoxia, ischemia, and infection, lead to dysfunction of various types of cells and to production of soluble mediators, resulting in wound underhealing or overhealing. Recently, several studies showed that local or systemic administration of stem or progenitor cells, such as mesenchymal stem cells (MSCs) and EPCs, enhances wound repair and angiogenesis [10, 21]. In particular, these studies revealed that the use of genetically engineered cell populations could increase the therapeutic efficacy, because the harsh pathological environment, including hypoxia, drastically affects the survival rate of the unmodified transplanted cells. In the present study, the major findings are as follows: (1) Lnk deficiency in EPCs enhances the expression of functional EPC markers and bioactivities such as proliferation, migration, and capacity for tubule-like formation; (2) a transplant of Lnk-deficient EPCs enhances wound repair via inhibition of recruitment of leukocytes in the inflammatory phase and by activation of myofibroblasts in the tissue-remodeling phase; (3) administration of Lnk-deficient EPCs improves wound healing in mice with STZ-induced diabetes.

Lnk is an adaptor protein that mediates protein–protein and protein–phospholipid interactions without an intrinsic enzymatic function [22, 23]. Lnk, as a key molecular target, augments the function of EPCs and neovascularization [12]. Our findings indicated that wound repair was significantly enhanced in Lnk-deficient mice as compared with wild-type mice. However, wound repair is a complex biological process that requires several cell types [1, 2], and the Lnk adaptor protein affects various of cell types, including hematopoietic stem cells [24], T cells [25], macrophages [26], EPCs [13, 14], and endothelial cells [27]. Therefore, to focus on one of the populations involved in the wound-healing process, we performed flow cytometry in the wound tissues to search for the recruitment of a specific cell population, and demonstrated that Lnk deficiency in mice specifically increased the recruitment of the EPC population to the injury sites. In a model of bone fracture, Lnk-deficient mice show improved osteogenesis because of enhanced angiogenesis through the recruitment of EPCs to the prefracture zone [28]. Our data also revealed that the EPC bioactivities, including proliferation, migration, and tube formation capacity were significantly higher in Lnk-deficient EPCs than in wild-type EPCs. Our previous studies showed that Lnk deficiency in mice promotes EPC kinetics and neovascularization in response to angiogenic cytokines, such as SCF, VEGF, and SDF-1 [13]. In addition, Lnk-deficient EPCs increase the clonogenic proliferation via activation of the JAK-STAT3 signal pathway [14]. These findings strongly support the notion that Lnk deficiency in mice promotes wound repair through the recruitment of EPCs and improves EPC cellular bioactivities, which are initiating steps of vascular repair, which is tightly regulated by the Lnk adaptor protein for cellular homeostasis.

EPCs, as key progenitors of endothelial cells, participate in neovascularization and tissue repair. After administration of a cutaneous wound to mice, BM-derived EPC mobilization is increased via the SDF-1α/CXCR4 axis [29]. A human cord blood-derived EPC transplant accelerates wound closure in nude mice with STZ-induced diabetes by stimulation of proliferation of keratinocytes and fibroblasts [30]. Our results show that wound closure is significantly better after an EPC transplant than after PBS injection. Moreover, the transplant of Lnk-deficient EPCs significantly enhanced wound healing through the improvement of transplanted-cell proliferation and survival as well as neovascularization, as compared with a transplant of wild-type EPCs. In a hindlimb ischemia model, our previous report clearly showed that a transplant of Lnk-deficient EPC enhances proliferation and survival as well as neovascularization through regulation of the JAK2/STAT3 signaling pathway [14]. In a mouse model of spinal cord injury, we also reported that Lnk-deficient, c-Kit-positive, Sca-1-positive, and lineage marker-negative cell populations (which are a core source of EPCs) enhance angiogenesis, astrogliosis, and functional recovery [19]. These previous reports support our present findings that a transplant of Lnk-deficient EPCs promotes wound healing through enhancement of neovascularization.

In the inflammatory phase, a healthy inflammatory reaction is involved in wound healing through the removal of necrotic tissues, debris, and pathogen contaminants, as well as via recruiting and activating fibroblasts. Leukocytes, including macrophages and neutrophils, appear in the wound at 1–3 days after injury and continue the process of phagocytosis [31]. Nonetheless, inflammation under pathological conditions such as in a chronic disease leads to delayed healing and promotes inflammation. In the proliferation phase (4–14 days after injury), overactivated immune cells induce scar formation and fibrosis [31, 32]. The possible reason for the continued presence of inflammatory cells is their persistent recruitment and activation due to tissue injury from enhanced mechanical pressure, pathogens, leukocyte trapping, and ischemic injury [20]. Cell death and tissue necrosis also cause inflammation [33]. In addition, inflammation and oxidative stress affect EPC mobilization. Our data show that neither wild-type nor Lnk-deficient EPCs affected immune cell recruitment in the inflammatory phase (postoperative day 3), whereas transplantation of Lnk-deficient EPCs significantly decreased the recruitment of macrophages and neutrophils after postoperative day 7 as compared with transplantation of wild-type EPCs. Human gingiva-derived MSCs accelerate wound healing by eliciting M2 polarization in macrophages [34]. In corneal injury, MSCs promote corneal wound healing by their anti-inflammatory action, including secretion of IL-10, IL-6, and transforming growth factor beta 1 (TGF-β1) [35]. IL-10-deficient EPCs show decreased survival and function at ischemic sites [36]. These results suggest that Lnk-deficient EPCs have an anti-inflammatory effect after a transplant in an excisional wound. Additional studies will obviously be necessary to further elucidate the complex role of Lnk-deficient EPCs in secretion of anti-inflammatory paracrine factors and their functions during dermal wound healing.

In the remodeling phase, activated fibroblasts, which have a myofibroblast phenotype, perform the ECM remodeling. Fibroblast-to-myofibroblast differentiation represents a pivotal process during wound healing and tissue repair, because the high contractile force generated by myofibroblasts is effective for physiological tissue remodeling [32, 37]. Although the mechanism of skin contraction is different between mice and humans, reduced fibroblast proliferation leads to a strong delay in wound closure [38]. To evaluate the potential preclinical and clinical application of Lnk-deficient EPCs as a cell-based therapeutic, we focused on the bioactivity of fibroblasts through transplantation of EPCs [31, 37, 39]. The results of this study indicate that Lnk-deficient EPCs activate fibroblasts in vitro and induce the differentiation of fibroblasts into myofibroblasts in vivo. In particular, the fibrotic area was significantly decreased in mice transplanted with Lnk-deficient EPCs compared with that of mice transplanted with wild-type EPCs. Myofibroblasts perform a key function in wound healing and in contractile forces [39]. Trophic activity of MSCs increases skin wound closure by activation of dermal fibroblasts [40]. Coculture of fibroblasts with EPCs improves functional recovery after a myocardial infarction [41]. Engraftment of EPCs into an excisional wound model in diabetic mice augments wound repair via fibroblast proliferation [30]. These findings indicate that Lnk-deficient EPCs may be engaged in a cross-talk with fibroblasts for wound healing, but the precise mechanism of action of Lnk-mediated signaling cascades and the difference in skin contraction between mice and humans should be further investigated to support their preclinical and clinical application.

Finally, we assessed the effect of a transplant of Lnk-deficient EPCs on wound repair in mice with STZ-induced diabetes to confirm the beneficial effects of Lnk-deficient EPCs in a chronic disease. Our results revealed that wound repair is significantly better after a transplant of Lnk-deficient EPCs as compared with that in other groups. This study has some limitations to comprehensively determine the effect of Lnk-deficient EPCs in chronic diseases in general. In particular, we established a type 1 diabetes mouse model for wound healing; however, a type 2 diabetes model might be a more appropriate chronic disease model. To reveal the availability and possibility of EPCs for cell-based therapy in diabetes, we first confirmed the effect of Lnk-deficient EPCs in a type 1 diabetes model. In addition, the wound-healing mechanism might be different between mice and humans, since skin contraction plays a greater role in the rodent wound-healing mechanism than in that of humans. Therefore, in future studies, the effect of *Lnk* gene silencing will be investigated in type 1 and 2 diabetic models, and the precise role of Lnk in EPC-mediated wound healing will be evaluated in the chronic disease condition to support preclinical and clinical application.

## Conclusions

Although the effects of Lnk deficiency in EPCs should be further studied in chronic disease models, our findings imply that Lnk-deficient EPCs might be a promising cell source for stem or progenitor cell-based treatments of chronic diseases. Taken together, this study involving murine disease models revealed that specific disruption of Lnk promotes EPC bioactivities such as proliferation, migration, and tube formation in vivo and in vitro*.* A transplant of Lnk-deficient EPCs promotes wound repair through enhancement of angiogenesis, inhibition of inflammation, and activation of myofibroblasts. In addition, our results showed the possibility of therapeutic application of *Lnk*-mutated EPCs in a diabetic wound model and give some clues to the precise regulation of the *Lnk* gene. Thus, specific targeting of Lnk-mediated signaling might be a promising strategy for the development of cell-based therapeutics.

## Abbreviations

BM: bone marrow
EBM: endothelial basal medium
EC: endothelial cell
ECM: extracellular matrix
EPC: endothelial progenitor cell
EPO: erythropoietin
ERK: extracellular signal-regulated kinase
FACS: fluorescence-activated cell sorter
H&E: hematoxylin and eosin
IL: interleukin
JAK: Janus kinase
MAPK: mitogen-activated protein kinase
MPL: myeloproliferative leukemia protein
MSC: mesenchymal stem cell
PCNA: proliferating cell nuclear antigen
PDGF: platelet-derived growth factor
PI3K: phosphoinositol-4,5-bisphosphate 3-kinase
SCF: stem cell factor
SDF-1: stromal cell-derived factor 1
SMA: smooth muscle actin
STAT3: signal transducer and activator of transcription
STZ: streptozotocin
TNF: tumor necrosis factor
TPO: thrombopoietin
VEGF: vascular endothelial growth factor
