# Erratum to: Specific disruption of Lnk in murine endothelial progenitor cells promotes dermal wound healing via enhanced vasculogenesis, activation of myofibroblasts, and suppression of inflammatory cell recruitment

**DOI:** 10.1186/s13287-017-0493-6

**Published:** 2017-03-09

**Authors:** Jun Hee Lee, Seung Taek Ji, Jaeho Kim, Satoshi Takaki, Takayuki Asahara, Young-Joon Hong, Sang-Mo Kwon

**Affiliations:** 1 Department of Pharmacology and Toxicology, University of Alabama at Birmingham School of Medicine, Birmingham, AL 35294 USA; 2 Department of Physiology, Laboratory for Vascular Medicine and Stem Cell Biology, Medical Research Institute, School of Medicine, Pusan National University, Yangsan, 626-870 Republic of Korea; 3 Research Institute of Convergence Biomedical Science and Technology, Pusan National University School of Medicine, Yangsan, Republic of Korea; 4 Department of Immune Regulation, Research Centre for Hepatitis and Immunology, Research Institute, National Centre for Global Health and Medicine, Chiba, Japan; 5 Department of Regenerative Medicine Science, Tokai University School of Medicine, Kanagawa, Japan; 6 Division of Cardiology of Chonnam National University Hospital, Cardiovascular Convergence Research Center Nominated by Korea Ministry of Health and Welfare, Gwangju, 501-757 Republic of Korea

## Erratum

Unfortunately, after publication of this article [1], it was noticed that Fig. 4 was incorrect. Panels B and D contained incorrect graphs. The corrected Fig. 4 can be seen below and the original article has been updated to correct this.

**Fig. 4:**
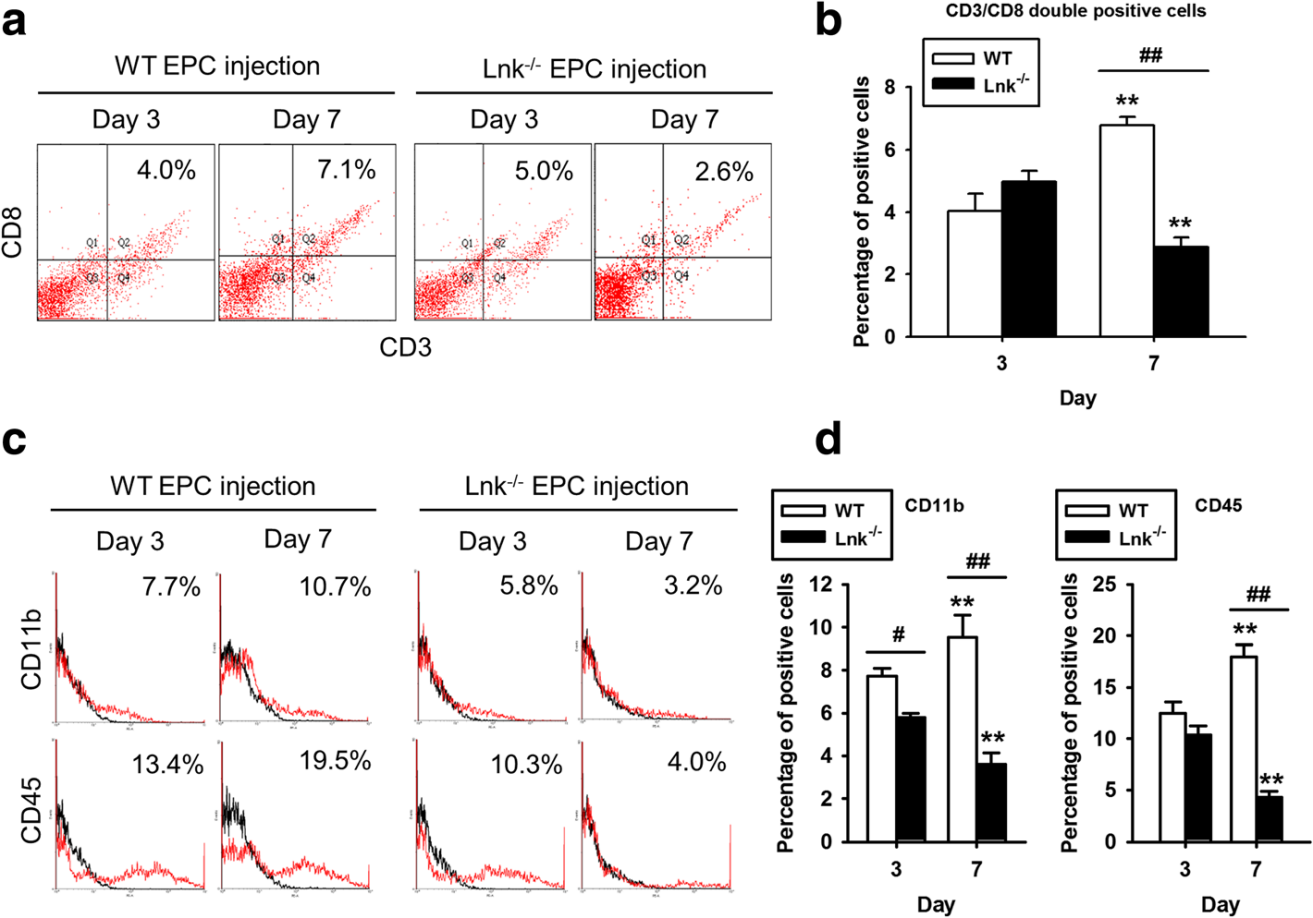
**A** transplant of Lnk-deficient EPCs suppresses the recruitment of inflammatory cells. After injection of wild-type (WT) and Lnk-deficient EPCs into wound sites, wound tissues were analyzed to determine the recruitment of cytotoxic T cells (CD3- and CD8-positive cells), macrophages (CD11b-positive cells), and neutrophils (CD45-positive cells) on postoperative days 3 and 7. **a** The recruitment of cytotoxic T cells in wound tissues was assessed by FACS analysis. **b** The percentage of CD3/CD8 double-positive cells on postoperative days 3 and 7. Values are mean ± SEM; **p < 0.01 compared to postoperative day 3, respectively, and ##*p <* 0.01 compared to injection with WT EPCs. **c** The recruitment of macrophages and neutrophils to wound tissues was assessed by FACS analysis. **d** The percentage of CD11b- and CD45-positive cells on postoperative days 3 and 7. Values are mean ± SEM;**p < 0.01 compared to postoperative day 3, respectively, #*p <* 0.05 and ##*p <* 0.01 compared to injection with WT EPCs]
